# Differences Between Randomized Clinical Trial Participants and Real-World Empagliflozin Users and the Changes in Their Glycated Hemoglobin Levels

**DOI:** 10.1001/jamanetworkopen.2019.20949

**Published:** 2020-02-07

**Authors:** Nicolai E. Munk, Jakob S. Knudsen, Anton Pottegård, Daniel R. Witte, Reimar W. Thomsen

**Affiliations:** 1 Department of Clinical Epidemiology, Institute of Clinical Medicine, Aarhus University Hospital, Aarhus, Denmark; 2 Clinical Pharmacology and Pharmacy, University of Southern Denmark, Odense, Denmark; 3 Department of Public Health, Aarhus University, Aarhus, Denmark; 4 Danish Diabetes Academy, Odense, Denmark; 5 Steno Diabetes Center Aarhus, Aarhus, Denmark

## Abstract

**Importance:**

Use of the sodium-glucose cotransporter 2 inhibitor empagliflozin has increased substantially since 2015. Little is known about characteristics of real-world patients who use empagliflozin or about empagliflozin’s effectiveness in reducing glycated hemoglobin (HbA_1c_) levels in routine clinical care.

**Objectives:**

To characterize real-world initiators of empagliflozin, to examine the proportion of initiators who would have been eligible for participation in phase 3 randomized clinical trials (RCTs) of empagliflozin, and to assess changes in HbA_1c_ levels after empagliflozin initiation.

**Design, Setting, and Participants:**

This cross-sectional study used linked population-based medical databases containing complete information on redeemed prescriptions, laboratory tests, and diagnoses for all residents in Northern Denmark. A total of 7034 residents of Denmark who filled a first-time empagliflozin prescription from January 2014 to December 2018 were included. Data analysis was performed in August 2019.

**Exposure:**

Empagliflozin initiation.

**Main Outcomes and Measures:**

Proportion of real-world users ineligible for RCT inclusion and absolute reduction in HbA_1c_ level 6 months after empagliflozin initiation.

**Results:**

Of 7034 first-time empagliflozin initiators (median [interquartile range] age, 61.50 [53.30-69.38] years; 4475 [63.6%] men), 3878 (55.1%) would have been ineligible for phase 3 RCT participation; frequent reasons were concurrent use of specific glucose-lowering drugs (1955 initiators [27.8%]), baseline HbA_1c_ level outside the eligibility range (1772 [25.2%]), or presence of comorbidities (1067 initiators [15.3%]). Initiation of empagliflozin was associated with a mean HbA_1c_ reduction of −0.91% (95% CI, −0.94% to −0.87%) after 6 months, similar to phase 3 RCT results. Real-world empagliflozin initiators who would have been eligible for RCT participation experienced slightly lower mean HbA_1c_ reductions (−0.78%; 95% CI, −0.82% to −0.74%) compared with patients who would have been ineligible (−1.01%; 95% CI, −1.07% to −0.95%). Ineligible initiators had higher median (interquartile range) baseline HbA_1c_ values than eligible initiators (8.5% [7.4% to 10.1%] vs 8.2% [7.6% to 9.8%]).

**Conclusions and Relevance:**

In this cross-sectional study, more than half of empagliflozin initiators exhibited clinical characteristics that would have led to ineligibility for the RCTs leading to the drug’s approval. While the findings suggest that the efficacy of empagliflozin in reducing HbA_1c_ levels translates into real-world effectiveness, further studies should examine clinical outcome effectiveness and drug safety in routine clinical care.

## Key Points

**Question:**

To what extent do real-world initiators of empagliflozin and the changes in their glycated hemoglobin (HbA_1c_) levels associated with empagliflozin use differ from participants and outcomes in randomized clinical trials?

**Findings:**

In this cross-sectional study of 7034 empagliflozin initiators, more than half would have been ineligible for the randomized clinical trials leading to the approval of empagliflozin, primarily because of baseline comedications, comorbidities, and HbA_1c_ levels. Overall, reductions in HbA_1c_ levels were similar between real-world patients and trial participants.

**Meaning:**

These findings suggest that the efficacy of empagliflozin in reducing HbA_1c_ in trials translates into real-world effectiveness, but they also underscore the importance of conducting studies after drug approval, given that real-world patients differ from randomized clinical trial participants.

## Teaser

This cross-sectional study characterizes real-world initiators of empagliflozin, examines the proportion of initiators who would have been eligible for participation in phase 3 randomized clinical trials of empagliflozin, and assesses changes in glycated hemoglobin levels after empagliflozin initiation.

## Introduction

For newer glucose-lowering drugs (GLDs), the real-world effectiveness in reducing glycated hemoglobin (HbA_1c_) levels may be different than the efficacy observed in randomized clinical trials (RCTs).^1^ This discrepancy may occur because real-world users of medications differ from RCT participants, eg, in terms of age, sex, comorbidities, disease severity, comedication, and duration and adherence to medication.^2,3^ Concerns among medical practitioners that the generalizability of RCT findings is limited have previously led to the underuse of new treatments proven efficacious by RCTs.^4^

Sodium-glucose cotransporter 2 inhibitors, most prominently empagliflozin,^5^ represent the newest class of GLDs. The sodium-glucose cotransporter 2 inhibitor empagliflozin was approved by the European Medical Agency and the US Food and Drug Administration in 2014 for the treatment of type 2 diabetes, based on 4 phase 3 RCTs.^6^ Following the publication of results from the EMPA-REG OUTCOME trial^7^ in 2015 and the publication of new guidelines, use of empagliflozin has increased substantially worldwide. Because sodium-glucose cotransporter 2 inhibitors, including empagliflozin, are among the newest GLD classes, little is known about their real-world use, effectiveness, and safety in routine clinical care.

In this study, we characterized new users of empagliflozin in Northern Denmark using prospectively collected data from Danish population-based medical databases. We examined GLD regimens used by empagliflozin initiators and their HbA_1c_ levels before and after empagliflozin initiation. We then compared the eligibility criteria of the 4 empagliflozin phase 3 RCTs with the characteristics of our study population.

## Methods

This registry-based study was approved by the Danish Data Protection Agency and needed no further ethics approval or patient consent according to Danish law. Thus, no informed consent was obtained from study participants, and there are no plans to involve patients in the dissemination of the results. This study followed the Strengthening the Reporting of Observational Studies in Epidemiology (STROBE) reporting guideline for cross-sectional studies.

### Setting and Study Population

We conducted a population-based sequential cross-sectional analysis in Northern Denmark (1.8 million residents, representing 32% of Denmark’s population) based on health care data from January 2009 to December 2018. The Danish National Health Service provides universal, tax-supported health care, guaranteeing unfettered access to general practitioners and hospitals and partial reimbursement for prescribed drugs. The Danish Civil Registration System was established in 1968 and provides daily updates of vital status and residency.^8^ The unique Civil Personal Registration number assigned to all Danish residents at birth or immigration makes unambiguous linkage of data sources at the individual level possible.^9^

Our study population included individuals who were residents of Northern Denmark for at least 1 year before starting a first-time empagliflozin prescription from January 2014 to December 2018. In Denmark, empagliflozin was approved for use among patients with a clinical diagnosis of type 2 diabetes during the study period. We linked existing population-based medical databases, including the Danish National Prescription Registry, the Danish National Patient Register (DNPR), and the Clinical Laboratory Information System (LABKA) database. The Danish National Prescription Registry covers all prescriptions redeemed at any pharmacy in Denmark starting in 1995.^10^ The DNPR contains individual-level information on dates of admission and discharge from all Danish nonpsychiatric hospitals starting in 1977 and records of emergency and outpatient specialist clinic visits starting in 1995.^11^ Each hospital encounter is recorded in the DNPR with a primary diagnosis and potentially multiple secondary diagnoses. The registry used the *International Classification of Diseases, Eighth Revision *(*ICD-8*) until the end of 1993 and the *ICD*-*10* thereafter. The LABKA database in Northern Denmark contains results from biochemistry tests ordered in primary care and hospitals, with complete coverage starting in 2000.^12^

### Characteristics of Empagliflozin Initiators

We used data prospectively collected before the date of empagliflozin initiation to create patient profiles and characterize their baseline status. For each patient, the latest HbA_1c_ measurement before empagliflozin initiation was obtained from the LABKA database. We used the following categories to categorize pretreatment HbA_1c_ levels: less than 6.5%, 6.5% to 6.9%, 7.0% to 7.4%, 7.5% to 7.9%, 8.0% to 8.9%, 9.0% to 9.9%, and 10% or greater (to convert to proportion of total hemoglobin, multiply by 0.01). Patient age was assessed at the first empagliflozin treatment initiation, available from the Civil Registration System database. We obtained information on comorbid conditions included in the Charlson Comorbidity Index (CCI)^11,13^ from all inpatient and outpatient hospital encounters (using both primary and secondary diagnoses) recorded in the DNPR before the first empagliflozin prescription. We computed the total CCI score for each patient (excluding diabetes) and defined 4 categories of comorbidity, as follows: total scores of 0 (no comorbidity), 1 (moderate comorbidity), 2 (severe comorbidity), and 3 or greater (very severe comorbidity). We separately assessed presence of hospital-diagnosed microvascular or macrovascular diabetes complications, obesity, cardiovascular disease drug use, and the most recent estimated glomerular filtration rate (eGFR) and low-density lipoprotein cholesterol value measured in primary or secondary care. We also examined and graphically displayed all GLD regimens besides empagliflozin used by patients in the study population for 1 year before and after the initial empagliflozin prescription.

### Trial Eligibility and HbA_1c_ Reduction

We evaluated a range of eligibility criteria from our health care databases. We examined current baseline GLD therapy at the time of empagliflozin initiation by assessing which GLD prescriptions empagliflozin initiators redeemed in the prior 120 days. In the phase 3 RCTs leading to marketing approval of empagliflozin, drugs that could be combined with empagliflozin included metformin,^14^ metformin with sulphonylureas,^15^ and pioglitazone with or without metformin.^16^ Age restrictions excluded patients younger than 18 years from participating. Restrictions on baseline HbA_1c_ levels excluded patients from participating in the phase 3 RCTs. Patients who received no GLD medication or therapy with trial GLDs 12 weeks before RCT participation could only participate in the RCTs if their last measured HbA_1c_ level was between 7.0% and 10.0%. In the phase 3 RCTs, efficacy in reducing HbA_1c_ was evaluated after 6 months of therapy. We estimated HbA_1c_ levels at 6 months after empagliflozin initiation by using the measurement recorded closest to day 180; only measurements recorded within 3 months of this date were included in the analysis. In the phase 3 RCTs, treatment with antiobesity drugs or systemic steroids 90 days before RCT participation excluded patients from participation. Real-world initiators who used such drugs 120 days before empagliflozin initiation were deemed ineligible for this study.

Several comorbid conditions led to exclusion from the phase 3 RCTs.^14,15,16,17^ We aimed to evaluate the prevalence of the following comorbid conditions in real-world initiators: body mass index (BMI, calculated as weight in kilograms divided by height in meters squared) greater than 45, liver diseases, renal diseases, acute coronary syndrome, transient ischemic attack or stroke within 3 months before empagliflozin initiation, cancer, bariatric surgery, uncontrolled endocrine disorders, blood dyscrasias, allergy to GLDs, plans for pregnancy or breastfeeding, alcohol or drug use disorders, mental incapacity, and any other clinical condition jeopardizing patients’ safety during RCT participation. We created an operational definition for each variable, adapted to the Danish National Prescription Registry,^10,11^ the DNPR,^11^ and the LABKA,^12^ as appropriate. We compared the characteristics of each real-world empagliflozin initiator with the eligibility criteria used in the 4 phase 3 RCTs^14,15,16,17^ (eTable 1 in the Supplement).

To provide a conservative estimate of the proportion of real-world initiators who would have been ineligible for the phase 3 RCTs, we used only exclusion criteria that were present in all RCTs (eTable 2 in the Supplement). For example, 1 phase 3 RCT^17^ excluded patients with basal cell carcinoma, but this exclusion criteria was not used in the other phase 3 RCTs.^14,15,16^ Consequently, this criterion was not applied in our study. An exception was eGFR; 1 phase 3 RCT^17^ excluded patients with eGFR values lower than 50 mL/min/1.73 m^2^, and the remaining phase 3 RCTs^14,15,16^ applied a lower cutoff value (ie, <30 mL/min/1.73 m^2^). In this case, we used eGFR values less than 50 mL/min/1.73 m^2^ as the cutoff value. When an RCT eligibility criterion was missing in our medical databases (ie, BMI, specific contraindications to study drug, uncontrolled endocrine disorders, or current participation in other RCTs), no patients were deemed ineligible based on that criterion. Patients were classified into 2 groups based on eligibility (ie, eligible and ineligible) to participate in the phase 3 RCTs.

### Statistical Analysis

We first calculated the proportion of the study population within the different age, sex, HbA_1c_ level, and comorbidity categories. We characterized GLD combination trends among patients, using all redeemed prescriptions within 3-month periods (ie, quarters) for 1 year before and after empagliflozin initiation, as described in more detail elsewhere.^18^ We calculated and plotted the proportion of patients in each category for each quarter. Patients initiating empagliflozin in 2018 were excluded because they did not have 1 year of follow-up data.

We assessed the number and proportion of real-world empagliflozin initiators fulfilling each eligibility criterion shared by the 4 phase 3 RCTs. We calculated the (geometric) mean HbA_1c_ levels at 6 months after empagliflozin initiation and computed the absolute (arithmetic) mean HbA_1c_ level reduction from baseline in percentage points with 95% CIs for all real-world initiators, separately for real-world initiators who were found eligible and ineligible for RCT participation, and separately for real-world initiators with specific baseline characteristics. Evaluating whether 95% CIs overlap conveys the same information as *P* < .05 as the threshold for statistical significance. This allowed us to examine how specific baseline patient characteristics were associated with empagliflozin use and reductions in HbA_1c_ levels. The statistical analysis was conducted in R version 3.3.2 (R Project for Statistical Computing).

## Results

### Patient Characteristics

A total of 7034 first-time users of empagliflozin in Northern Denmark during the period from January 2014 to December 2018 were identified. The participants’ median (interquartile range [IQR]) age at empagliflozin initiation was 61.50 (53.30-69.38) years; 4475 (63.6%) were men (Table). Among them, 3878 (55.1%) would have been ineligible for phase 3 RCT participation. The proportion of initiators with any comorbidities (ie, CCI score >0) was higher among patients ineligible for the RCTs than those eligible for the RCTs (2362 of 3878 [60.9%] vs 1574 of 3156 [49.9%]) (Table). Diabetes-related complications were also more common in the ineligible group (847 [21.8%] vs 613 [19.4%]), and their median (IQR) age was slightly higher (62.55 [53.90-70.20] years vs 60.50 [52.60-68.30] years (Table). Median (IQR) duration of diabetes at empagliflozin start was similar for ineligible and eligible initiators (8.00 [4.20-12.40] years vs 7.85 [4.40-12.33] years) (Table). Ineligible initiators had higher median (IQR) baseline HbA_1c_ values than eligible initiators (8.5% [7.4%-10.1%] vs 8.2% [7.6%-9.8%]).

**Table.** Baseline Characteristics of 7034 Real-World Empagliflozin Initiators in Northern Denmark, 2014 to 2018

Characteristic	No. (%)		
	Ineligible Group (n = 3878)	Eligible Group (n = 3156)	Total (N = 7034)
Sex			
Men	2401 (61.9)	2074 (65.7)	4475 (63.6)
Women	1477 (38.1)	1082 (34.3)	2559 (36.4)
Age, y			
Median (IQR)	62.55 (53.90-70.20)	60.50 (52.60-68.30)	61.50 (53.30-69.38)
<50	611 (15.8)	533 (16.9)	1164 (16.5)
50 to <60	1040 (26.8)	965 (30.6)	2005 (28.5)
60 to <70	1231 (31.7)	1009 (32.0)	2240 (31.8)
70 to <80	834 (21.5)	550 (17.4)	1384 (19.7)
≥80	162 (4.2)	79 (2.5)	241 (3.4)
Diabetes duration			
Median (IQR), y	8.00 (4.20-12.40)	7.85 (4.40-12.33)	7.90 (4.30-12.40)
<3 mo	118 (3.0)	77 (2.4)	195 (2.8)
3 mo to <1 y	303 (7.8)	222 (7.0)	525 (8.3)
1-3 y	396 (10.2)	313 (9.9)	709 (10.1)
3-5 y	441 (11.4)	357 (11.3)	798 (11.3)
≥5 y	2738 (70.6)	2264 (71.7)	5002 (71.1)
Any hospital diagnosis of microvascular complications	847 (21.8)	613 (19.4)	1460 (20.8)
Neurological complication	244 (6.3)	165 (5.2)	409 (5.8)
Ocular complication	847 (21.8)	613 (19.4)	1460 (20.8)
Renal complication	204 (5.3)	103 (3.3)	307 (4.4)
Hospital diagnosis of obesity	1017 (26.2)	780 (24.7)	1797 (25.5)
Hospital diagnosis of ASCVD	1361 (35.1)	938 (29.7)	2299 (32.7)
CCI score^a^			
0	1516 (39.1)	1582 (50.1)	3098 (44.0)
1	427 (11.0)	363 (11.5)	790 (11.2)
2	836 (21.6)	691 (21.9)	1527 (21.7)
≥3	1099 (28.3)	520 (16.5)	1619 (23.0)
eGFR			
<60 mL/min/1.73 m^2^	380 (9.8)^b^	193 (6.1)	570 (8.1)^b^
>60 mL/min/1.73 m^2^	3494 (90.1)^b^	2954 (93.6)	6450 (91.7)^b^
No measurements	0	9 (0.3)	9 (0.1)
CVD drug use			
Statin	2927 (75.5)	2487 (78.8)	5414 (77.0)
Any antihypertensives	3315 (85.5)	2694 (85.4)	6009 (85.4)
ACE inhibitor	1447 (37.3)	1297 (41.1)	2744 (39.0)
LDL cholesterol			
Median (IQR), mg/dL	73.4 (57.9-100.4)	73.4 (54.1-92.7)	73.4 (54.1-96.5)
No measurement	20 (0.5)	21 (0.7)	41 (0.6)
Baseline HbA_1c_ level, %			
Median (IQR)	8.5 (7.4-10.1)	8.2 (7.6-9.8)	8.3 (7.6-9.4)
<6.5	200 (5.2)	0	200 (2.8)
6.5-6.9	515 (13.3)	0	515 (7.3)
7.0-7.4	365 (9.4)^b^	568 (18.0)^b^	933 (13.3)^b^
7.5-7.9	452 (11.7)	680 (21.5)	1132 (16.1)
8.0-8.9	797 (20.6)	1205 (38.2)	2002 (28.5)
9.0-9.9	483 (12.5)	690 (21.9)^b^	1174 (16.7)^b^
≥10	1060 (27.3)^b^	<5 (0.2)^b^	1058 (15.0)^b^
No measurements	9 (0.2)	11 (0.3)	20 (0.3)

Abbreviations: ACE, angiotensin-converting enzyme; ASCVD, atherosclerotic cardiovascular disease; CCI, Charlson Comorbidity Index; CVD, cardiovascular disease; eGFR, estimated glomerular filtration rate; HbA_1c_, glycated hemoglobin; IQR, interquartile range; LDL, low-density lipoprotein.

SI conversion factors: To convert LDL cholesterol to millimoles per liter, multiply by 0.0259; HbA_1c_ to proportion of total hemoglobin, multiply by 0.01.

^a^ The CCI includes 19 major disease categories, ascertained from each individual’s complete hospital contact history before the date of initial empagliflozin treatment. Diabetes was excluded from CCI scores.

^b^ Danish law requires reporting of approximate counts in some cases where low numbers (ie, n < 5) may be inferred from the counts in other categories; thus, these data are approximations.

**Figure 1.**
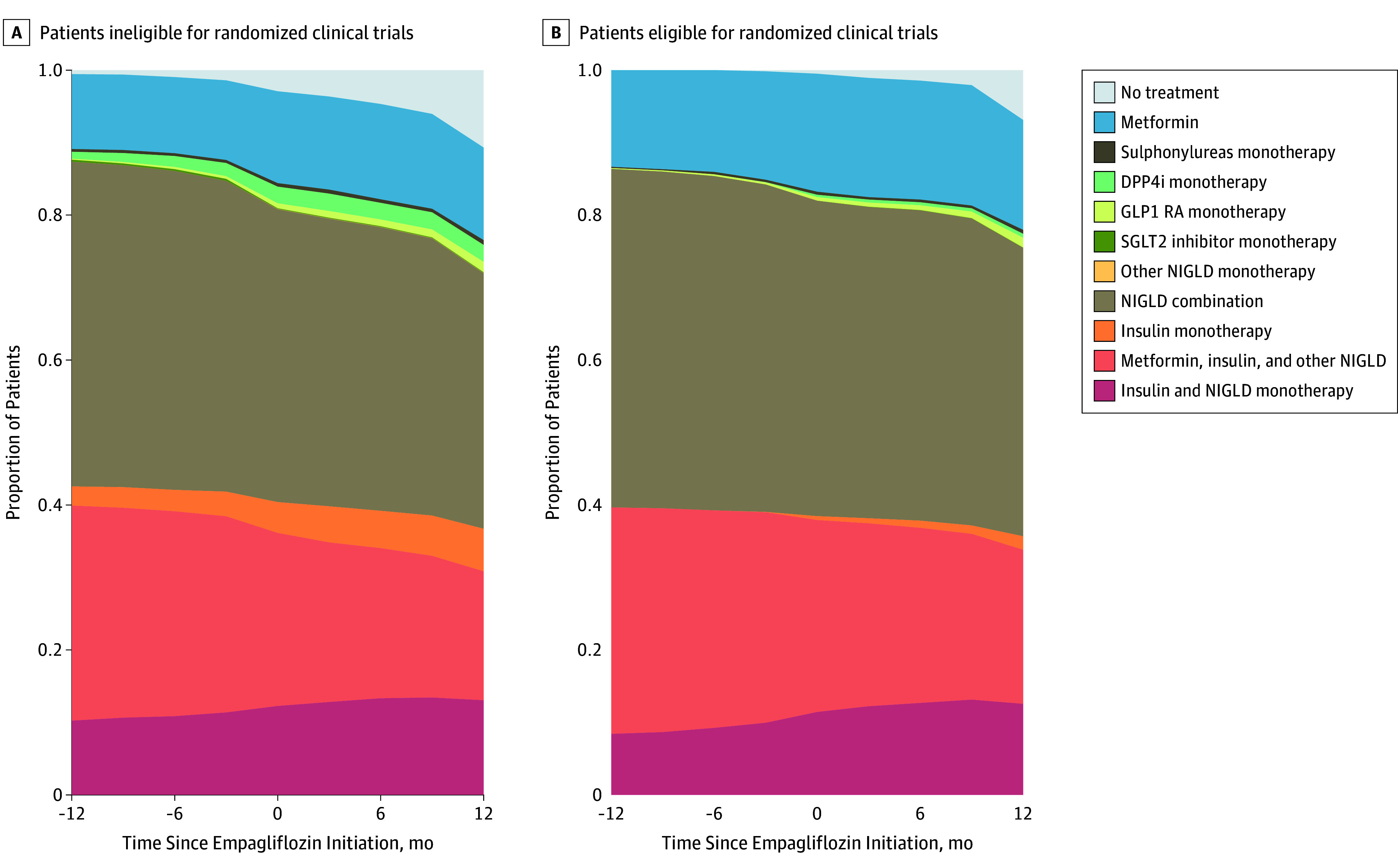
Glucose-Lowering Drug Regimens Besides Empagliflozin Used Within 1 Year Before and After Empagliflozin Initiation The figure depicts the proportion of patients ineligible (A) and eligible (B) for empagliflozin randomized clinical trials who used other glucose-lowering drug treatments. Empagliflozin prescriptions were excluded. DPP4i indicates dipeptidyl peptidase-4 inhibitor; GLP1 RA, glucagon-like peptide-1 receptor agonist; NIGLD, noninsulin glucose-lowering drug; and SGLT2, sodium-glucose cotransporter 2.

In general, GLD use and trends before and after empagliflozin initiation were similar between eligible and ineligible initiators (Figure 1). The most common GLD regimens used besides empagliflozin in the 3 months after empagliflozin initiation were either noninsulin GLD combination therapy alone (2401 initiators [34.1%]) or with metformin and insulin (1071 initiators [15.2%]). However, the proportion of initiators who received metformin or at least 2 other noninsulin GLDs decreased from 1266 initiators to 935 initiators after empagliflozin initiation, indicating that a number of patients used empagliflozin as replacement therapy rather than augmentation therapy (Figure 1).

### Trial Eligibility and HbA_1c_ Reduction With Empagliflozin Therapy

**Figure 2.**
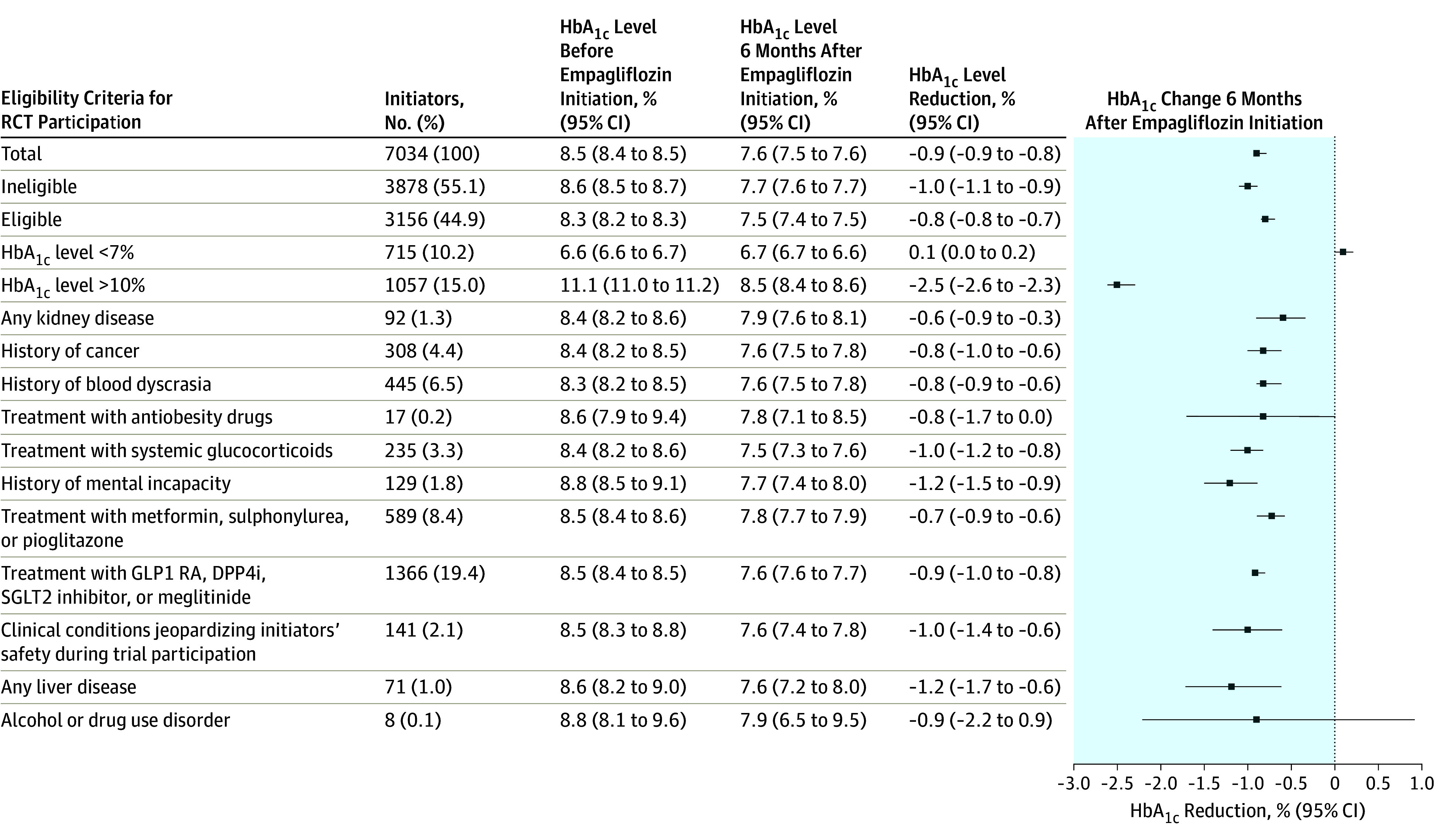
Real-World Empagliflozin Initiators Who Would Have Been Excluded From Randomized Clinical Trials (RCTs) and Their Glycated Hemoglobin (HbA_1c_) Level Change DPP4i indicates dipeptidyl peptidase-4 inhibitor; GLP1 RA, glucagon-like peptide-1 receptor agonist; and SGLT2, sodium-glucose cotransporter 2. To convert HbA_1c_ to proportion of total hemoglobin, multiply by 0.01.

Figure 2 shows the overall proportion of real-world empagliflozin initiators assessed to be ineligible for RCT participation. It also shows these patients stratified by each eligibility criterion and their HbA_1c_ measurements at baseline and after 6 months.

Overall, 3878 initiators (55.1%) would have been ineligible for RCT participation (Table and Figure 2). The main reasons for RCT ineligibility were noneligible GLD comedications before empagliflozin initiation, occurring in a total of 1955 initiators (27.8%); of these, 1366 initiators (19.4%) received therapy with glucagon-like peptide-1 receptor agonists, dipeptidyl peptidase-4 inhibitors, a sodium-glucose cotransporter 2 inhibitor other than empagliflozin, or meglitinide. A total of 1772 initiators (25.2%) had HbA_1c_ levels outside the range required for RCT participation; of these, 1057 initiators (15.0%) had HbA_1c_ levels greater than 10.0% and 715 initiators (10.2%) had HbA_1c_ levels less than 7.0% (Figure 2).

A total of 1067 real-world empagliflozin initiators (15.3%) would have been ineligible for RCT participation because of severe comorbidities, such as blood dyscrasias (eg, anemia or coagulation defects) (445 [6.5%]), cancer (308 [4.4%]), or other clinical conditions jeopardizing initiators’ safety, such as esophageal varices, recent endocarditis, or thoracic aortic surgery (141 [2.1%]) (Figure 2).

Overall, initiation of empagliflozin was associated with a mean HbA_1c_ reduction of −0.91% (95% CI, −0.94% to −0.87%) within 6 months. It was slightly greater among ineligible initiators (−1.01%; 95% CI, −1.07% to −0.95%) than among eligible initiators (−0.78%; 95% CI, −0.82% to −0.74%). Mean baseline HbA_1c_ levels were higher among ineligible initiators (8.60%; 95% CI, 8.55% to 8.66%) than among eligible initiators (8.26%; 95% CI, 8.24% to 8.29%) (Figure 2). The 4 phase 3 RCTs found a similar pooled adjusted mean HbA_1c_ reduction of −0.74% (95% CI, −0.75% to −0.73%) after 6 months compared with the mean reduction in the placebo groups (−0.12% reduction).^6^ All patients in the 4 phase 3 RCTs had lower mean baseline HbA_1c_ (range, 7.87% to 8.10%) compared with real-world patients in the present study.^14,15,16^

An important difference between the ineligible cohort and the eligible cohort was the possibility of lower (ie, <7.0%) and higher (ie, >10.0%) baseline HbA_1c_ levels among ineligible initiators. Thus, 715 of 3878 ineligible initiators (18.4%) had HbA_1c_ levels lower than 7.0% at baseline, while this proportion was, by definition, 0% among eligible initiators. Individuals with baseline HbA_1c_ levels less than 7.0% experienced very minor mean increases in HbA_1c_ levels after empagliflozin initiation (0.10%; 95% CI, 0.04% to 0.17%). When all initiators with baseline HbA_1c_ levels of less than 7.0% were excluded from the ineligible cohort, a mean HbA_1c_ reduction of −1.25% (95% CI, −1.31% to −1.18%) was observed in the ineligible cohort.

## Discussion

This cross-sectional study found that more than half of individuals in Northern Denmark who started using empagliflozin from 2014 to 2018 would have been ineligible for participation in phase 3 RCTs of empagliflozin. These exclusions were predominantly because of concurrent use of other GLDs and HbA_1c_ levels outside RCT eligibility criteria. In a similar 2018 study in our region,^19^ 3 of 4 real-world liraglutide initiators from 2009 to 2015 would have been ineligible for any phase 3 RCT that led to the drug’s marketing approval.^20^ Real-world liraglutide initiators were more often ineligible for RCTs because of comorbidities than real-world empagliflozin initiators because the liraglutide RCTs had stricter comorbidity eligibility criteria than the empagliflozin RCTs.^6^

Overall, reductions in HbA_1c_ levels were of similar magnitude among real-world empagliflozin initiators, including initiators who would have been ineligible for the RCTs, and RCT participants (Figure 2). However, our real-world patients occasionally applied empagliflozin as a replacement therapy rather than as an add-on therapy, an approach that was not included in the corresponding RCTs. This could have biased our findings toward an underestimation of the real-world reduction in HbA_1c_ levels compared with the reduction seen in RCTs. The subgroup of ineligible initiators experienced a greater HbA_1c_ reduction than RCT participants. Real-world patients with lower baseline HbA_1c_ levels experienced less reduction in HbA_1c_ levels than RCT participants.

Patients enrolled in phase 3 RCTs likely exhibited healthier behavior, including higher medication adherence, and were encouraged to tolerate more adverse effects than real-world users. A 2017 study^1^ found a 0.8% larger absolute reduction in HbA_1c_ values with glucagon-like peptide-1 receptor agonist therapy among RCT participants (ie, −1.3%) compared with real-world users (ie, −0.5%). The authors concluded that poor adherence was the primary reason for reduced real-world effectiveness of glucagon-like peptide-1 receptor agonists. While drug adherence is not directly addressed in our study, some exclusion criteria for the RCTs did address a patient’s ability to adhere to the trial regimen (eg, uncontrolled hypoglycemia, drug and alcohol use disorders, and mental incapacity) to ensure selection of a highly adherent RCT population. This allowed an effect to be detected in an intention-to-treat analysis.^21^ While this is an understandable approach, differences in comorbidities could imply the possible presence of lower effectiveness of empagliflozin, more adverse effects, and unknown adverse effects in real-world users compared with RCT participants. We did not find a smaller reduction in HbA_1c_ levels among our real-world empagliflozin initiators. While the present study appears to have established the glucose-lowering effectiveness of empagliflozin, safety data are still sparse for empagliflozin initiators outside RCTs.

### Strengths and Limitations

Major strengths of our study include the use of population-based, high-validity registries.^11^ The Danish comprehensive public health care system in a well-defined geographical region eliminates some of the selection problems seen in clinic-based studies, and the large study size provided precise estimates of HbA_1c_ levels. In Denmark, all reimbursed drug prescriptions have to be redeemed at monopolized community pharmacies, leading to complete coverage of empagliflozin prescriptions in our study.^10,22^

Because of the limitations of administrative data, some RCT criteria were not available for evaluation (eg, BMI outside range or uncontrolled endocrine disorders). Considering that these conditions may be frequent, we may have underestimated the proportion of ineligible patients in our study. Moreover, although empagliflozin was only approved for type 2 diabetes, we may have included some patients in our study who did not have true type 2 diabetes because of missing clinical and biochemical data (eg, antibodies) on exact diabetes type.

## Conclusions

Our findings suggest that many real-world empagliflozin initiators have higher or lower baseline HbA_1c_ levels or initiate empagliflozin with other GLDs compared with patients enrolled in the phase 3 RCTs that led to the marketing approval of empagliflozin. Nevertheless, the observed efficacy of empagliflozin in reducing HbA_1c_ levels seen in the RCTs appeared to translate into a real-world association of empagliflozin use and reductions in HbA_1c_ levels among both eligible and ineligible empagliflozin initiators. Further studies should examine clinical outcome effectiveness and drug safety in these patient groups in routine clinical care.

## Notes

Supplement. **eTable 1. **Definition of Eligibility Criteria for Empagliflozin RCTs **eTable 2. **Real-World Empagliflozin Initiators Who Would Have Been Excluded From RCTs and Changes in Their Glycated Hemoglobin Levels
