# Supplementary material for: Differences Between Randomized Clinical Trial Participants and Real-World Empagliflozin Users and the Changes in Their Glycated Hemoglobin Levels
Source: JAMA Netw Open. 2020 Feb 7;3(2):e1920949. doi: 10.1001/jamanetworkopen.2019.20949 (PMC12549138; doi:10.1001/jamanetworkopen.2019.20949)
Supplement: Supplement. — eTable 1. Definition of Eligibility Criteria for Empagliflozin RCTs eTable 2. Real-World Empagliflozin Initiators Who Would Have Been Excluded From RCTs and Changes in Their Glycated Hemoglobin Levels [file jamanetwopen-e1920949-s001.pdf]

Supplementary Online Content

Munk NE, Knudsen JS, Pottegård A, Witte DR, Thomsen RW. Differences between randomized clinical trial participants and real-world empagliflozin users and the changes in their gylcated hemoglobin levels. *JAMA Netw Open*. 2020;3(2):e1920949. doi:10.1001/jamanetworkopen.2019.20949

eTable 1. Definition of Eligibility Criteria for Empagliflozin RCTs
eTable 2. Real-World Empagliflozin Initiators Who Would Have Been Excluded From RCTs and Changes in Their Glycated Hemoglobin Levels

This supplementary material has been provided by the authors to give readers additional information about their work.

© 2020 Munk NE et al. *JAMA Network Open.*

eTable 1. Definition of Eligibility Criteria for Empagliflozin RCTs

RCTs ineligibility common ground	Definition in Danish medical databases	Databases	Codes
Inclusion A.<br>≥18 years of age	Age at first empagliflozin prescription	The Danish Civil Registration System	
Inclusion B.<br>Pre-treated with non-insulin (ni)-GLDs for 3 months	≥1 ni-GLD prescriptions received any time, with the most recent prescription <120 days before empagliflozin start	The Danish National Prescription Registry	ATC<br>"A10B"
Inclusion C.<br>Glycated haemoglobin of ≥7% and ≤10% at visit 1 (screening) in order to be eligible for randomized treatment	Last glycated haemoglobin value measured before empagliflozin prescription. Patients on ni-GLD or no therapy: Glycated haemoglobin level 7-10%	LABKA	
Inclusion D.<br>BMI≤ 45	No data available		
Exclusion 2.<br>No other antidiabetic drug within 12 weeks prior to randomization except those mentioned in inclusion criteria	≥1 ni-GLD prescriptions received any time, with the most recent prescription <180 days before empagliflozin start of:<br>Exclusion if received the following drugs <180 days before empagliflozin prescription:<br>Meglitinide (repaglinide)<br>or gliptins (linagliptin, saxagliptin, sitagliptin, vildagliptin) or<br>SGLT2 (canagliflozin, dapagliflozin, ertugliflozin) or<br>GLP-1-analogs (exenatide, lixisenatide, semaglutide, dulaglutide, Insulin glargine, insulin degludec)	The Danish National Prescription Registry	ATC<br><br>"A10BX02",<br>"A10BH01",<br>"A10BH02",<br>"A10BH03",<br>"A10BH04",<br>"A10BH05"<br><br>"A10BK02",<br>"A10BK01",<br>"A10BK04",<br>"A10BJ01", "A10Bj03",<br>"A10BJ06","A10AE54",<br>"A10BJ05", "A10AE56"
Exclusion 3.<br>No Indication of liver disease, defined by serum levels of either ALT (SGPT), AST (SGOT), or alkaline phosphate above 3 x upper limit (U/L) of normal as determined during screening and/or run-in phase.	Last value carried forward to fist empagliflozin prescription:<br>Women: ALAT >135 U/L<br>Men: ALAT >210 U/L<br>All: basic phosphatase >315 U/L	LABKA	
Exclusion 4:<br>No acute coronary syndrome (ACS), transient ischemic attack (TIA) or stroke within 3	Within 3 months (before first empagliflozin prescription):<br><br><br>ACS:<br>"I21", "I22", "I23", "I200"	Danish National Patient Registry	Within 3 months (before first empagliflozin prescription):<br>"DI21"," DI22", "DI23",<br>"DI200", "DI60",

© 2020 Munk NE et al. *JAMA Network Open.*

eTable 1. Definition of Eligibility Criteria for Empagliflozin RCTs

RCTs ineligibility common ground	Definition in Danish medical databases	Databases	Codes
months prior to informed consent.	Stroke:<br>"I60-I69", "G45", "G46"<br>Intracranial endovascular thrombolysis or thrombectomy:<br>"KAAL10", "KAAL11"<br>TCI: "G45"		"DI61", "DI62", "DI63", " DI64", "DG45"<br>"KAAL10", "KAAL11",<br>"DG45"
Exclusion 5:<br>No impaired renal function, defined as (eGFR <50ml/min) as determined during screening and/ or run-in phase.	Last value carried forward:<br>eGFR <50ml/min	LABKA	
Exclusion 6.<br>No bariatric surgery within the past two years and other GI surgeries that induce chronic malabsorption	Within 2 years prior to first empagliflozin prescription:<br>Gastric bypass:<br>"KJDF10"<br>Laparoscopic gastric bypass:<br>"KJDF11"<br>Gastric banding:<br>"KJDF20"<br>Laparoscopic gastric banding:<br>"KJDF21"<br>Gastric sleeve:<br>"KJDF40"<br>Laparoscopic gastric sleeve:<br>"KJDF41"	Danish National Patient Registry	Within 2 years prior to first empagliflozin prescription.<br>"KJDF10", "KJDF11"<br>"KJDF20", "KJDF21"<br>"KJDF40", "KJDF41"
Exclusion 7.<br>No medical history of cancer (except for BCC) and/ or treatment for cancer within the last 5 years.	Hospital diagnoses of cancer within 5 years from first empagliflozin prescription:<br>Neoplasms:<br>"C0-C96"<br>Except: "C44" BCC	Danish National Patient Registry	Within 5 years from first empagliflozin prescription:<br>Neoplasms:<br>"DC0-DC096"<br>Except: "DC44"
Exclusion 8.<br>No blood dyscrasias or any other disorder hemolysis or unstable red blood cells (e.g. malaria, babesiosis, hemolytic anemia)	Ever before:<br>Polycythemia vera: "D45"<br>Myelodysplastic syndrome: "D46"<br>Histiocytic and mast cell tumours of uncertain and unknown behavior: "D47"<br>Nutritional deficiency anemia: "D50-D53"<br>Hemolytic anemia: "D55-D59"<br>Aplastic and other anemia: "D60-64"<br>Coagulation defects, purpura and other hemolytic conditions: "D65-D69"<br>Agranulocytosis: "D70"<br>Functional disorders of polymorphonuclear neutrophils: "D71"<br>Other disorders of white blood cell: "D72"<br>Methaemoglobinaemia: "D74"	Danish National Patient Registry	Ever before:<br>"DD45", "DD46",<br>"DD47", "DD50",<br>"DD51", "DD52",<br>"DD53", "DD55",<br>"DD56", "DD57",<br>"DD58", "DD59",<br>"DD60", "DD61",<br>"DD63", "DD64",<br>"DD65", "DD66",<br>"DD67", "DD68",<br>"DD69", "DD70",<br>"DD71", "DD72",<br>"DD74", "DD75",<br>"DD80", "DD81",<br>"DD82","DD83",<br>"DD84", "DB50",<br>"DB51", "DB52",<br>"DB53", "DB54",

© 2020 Munk NE et al. *JAMA Network Open.*

eTable 1. Definition of Eligibility Criteria for Empagliflozin RCTs

RCTs ineligibility common ground	Definition in Danish medical databases	Databases	Codes
	Other diseases of blood and blood-forming organs: ”D75”<br>Immunodeficiency with predominantly antibody defects: ”D80”<br>Combined immunodeficiency: ”D81”<br>Immunodeficiency associated with other major defects: ”D82”<br>Common variable immunodeficiency: ”D83”<br>Other immunodeficiencies: ”D84”<br>Plasmodium falciparum malaria: ”B50-B54”<br>Congenital malaria: ”P37.3”, ”P37.4”<br>Babesiosis: ”B60.0”		“DP373”, “DP374”, “DB600”
Exclusion 9.<br>No contraindications to metformin, empagliflozin, sulfonylurea or pioglitazone	No data available		
Exclusion 10.<br>No treatment with anti-obesity drug 3 months prior to informed consent	≥1 anti-obesity drug prescription received any time, with the most recent prescription <120 d before empagliflozin initiation:<br>Orlistat<br>Amfepramon	The Danish National Prescription Registry	ATC<br>“A08AB01”<br>“A08AA03”
Exclusion 11:<br>No females of child bearing potential who are pregnant, breast-feeding or have the intention of becoming pregnant or not using adequate contraceptive methods	Child birth ≤1 year before empagliflozin therapy or 1 year after:<br>Preterm labour without delivery: “O60”<br>Failed induction of labour: ”O61”<br>Abnormalities of forces of labour: ”062”<br>Long labour: ”O63”<br>Obstructed labour due to malposition and malpresentation of fetus: ”O64-O69”, ”O75”<br>Perineal laceration during delivery: ”O70”<br>Other obstetric trauma: ”O71”<br>Postpartum haemorrhage: ”O72”<br>Retained placenta and membranes, without haemorrhage: ”073”<br>Complications of anesthesia during labour and delivery: ”O74”<br>Single spontaneous delivery: ”O80-O83”<br>Multiple delivery: ”O84”<br>Obstetric embolism: ”O88”	Danish National Patient Registry	”DO60”, “DO61”, “DO62”, “DO63”, “DO64”, “DO65”, “DO66”, “DO67”, “DO68”, “DO69”, “DO75”, “DO70”, “DO71”, “DO72”, “DO73”, “DO74”, “DO80”, “DO81”, “DO82”, “DO83”, “DO84”, “DO88”
Exclusion 12:<br>No current treatment with systemic steroids at time of	≥1 prescription for oral glucocorticoids (prednisolone etc.) ≤120 days before empagliflozin therapy	The Danish National Prescription Registry	ATC<br>“H02AB”

eTable 1. Definition of Eligibility Criteria for Empagliflozin RCTs

RCTs ineligibility common ground	Definition in Danish medical databases	Databases	Codes
informed consent or change in dosage of thyroid hormones within 6 weeks prior to informed consent.			
Exclusion 13.<br>No participation in another trial with an investigational drug within 30 days prior to informed consent.	Not appropriate		
Exclusion 14.<br>No alcohol or drug abuse within the 3 months prior to informed consent that would interfere with trial participation or any ongoing condition leading to a decreased compliance to study procedures or study drug intake	Within 3 months<br>Mental and behavioral disorders due to psychoactive substance use:<br>“F10-F19”<br><br>Problems related to lifestyle:<br>Drug use: ”Z72.2”<br>Abuse of antidepressants: ”F550”<br>Abuse of laxatives: ”F551”<br>Abuse of antacids: ”F553”<br>Abuse of non-dependence-producing substances: ”F55”<br>Abuse of other non-psychoactive substances: ”F552”<br>Abuse of steroids or hormones: ”F555”<br><br>Alcohol related:<br>Degeneration of nervous system due to alcohol: ”G31.2”<br>Alcoholic polyneuropathy: ”G62.1”<br>Alcoholic myopathy: ”G72.1”<br>Alcoholic cardiomyopathy: ”I42.6”<br>Alcoholic gastritis: ”K292”<br>Alcohol-induced chronic pancreatitis: ”K860”<br>Alcoholic liver disease: ”K70”<br>Toxic effect of alcohol: ”T51”<br>Alcohol abuse counselling and surveillance: ”Z71.4”<br>Alcohol use: ”Z72.1”	Danish National Patient Registry	Within 3 months<br>Mental and behavioral disorders due to psychoactive substance use:<br>“DF10”, ”DF11”,<br>“DF12”, ”DF13”,<br>“DF14”, ”DF15”,<br>“DF16”, ”DF17”,<br>“DF18”, ”DF19”,<br><br>Problems related to lifestyle:<br>“DZ722”, ”DF550”,<br>”DF551”, ”DF553”,<br>”DF55”, ”DF552”,<br>”DF555”<br><br>Alcohol related:<br>”DG312” ”DG621”<br>”DG721” ”DI426”<br>”DK292” ”DK860”<br>”DK70” ”DR780”<br>”DT51” ”DZ714”<br>”DZ721”
Exclusion 15.<br>No receipt of any investigational drug	Not appropriate		

© 2020 Munk NE et al. *JAMA Network Open.*

eTable 1. Definition of Eligibility Criteria for Empagliflozin RCTs

**RCTs ineligibility common ground**	**Definition in Danish medical databases**	**Databases**	**Codes**
with four weeks to this trial			
Exclusion 16.<br>No uncontrolled endocrine disorder	Not appropriate		
Exclusion 17.<br>Any other clinical condition that could jeopardize patient safety while participating in this trial	Within 3 months (before first empagliflozin prescription):<br><br>Ligature of intracranial aneurysm and other intracranial vascular lesion:<br>”AAC”<br><br>Surgery of cranial and intracranial lesions:<br>”AAD”<br><br>Surgery of the major thoracic veins:<br>Suture or repair of thoracic vena cava: “KFAA”<br>Incision and excision of thoracic vena cava: “KFAB”<br>Resection, reconstruction or ligature of thoracic vena cava: “KFAC”<br>Bypass operations on thoracic vena cava: “KFAD”<br>Operations subsequent to cavo-pulmonary connection: “KFAF”<br>Other operations on major thoracic veins: “KFAW”<br><br>Pulmonary artery with branches:<br>Suture or repair of pulmonary artery: “FBA”<br>Incision of pulmonary artery: “FBB”<br>Banding operations on pulmonary artery: “FBC”<br>“Debanding operations pulmonary artery: “FBD”<br>Expansion and reconstruction of pulmonary artery: “FBE”<br>Reduction of pulmonary artery: “FBF”	Danish National Patient Registry	Within 3 months (before first empagliflozin prescription):<br><br>Ligature of intracranial aneurysm and other intracranial vascular lesion:<br>“KAAC00”, “KAAC05”, “KAAC10”, “KAAC15”, “KAAC20”, “KAAC30”, “KAAC40”, “KAAC45”, “KAAC99”<br><br>Surgery of cranial and intracranial lesions:<br>”KAAD00”, ”KAAD05”, ”KAAD10”, ”KAAD15”, ”KAAD99”<br><br>Surgery of the major thoracic veins:<br>”KFAA”, ”KFAB”, ”KFAC”, ”KFAD”, ”KFAF”, ”KFAW”<br><br>Pulmonary artery with branches:<br>”KFBA”, ”KFBB”, ”KFBC”, ”KFBD”, ”KFBE”, ”KFBF”, ”KFBG”, ”KFBH”, ”KFBJ”, ”KFBK”, ”KFBL”, ”KFBM”, ”KFBN”, ”KFBW”

eTable 1. Definition of Eligibility Criteria for Empagliflozin RCTs

RCTs ineligibility common ground	Definition in Danish medical databases	Databases	Codes
	Unifocalization in MAPCA with implantation in pulmonary artery or graft: “FBG”<br>Repair of pulmonary artery sling: “FBH”<br>Connection to pulmonary artery from subclavian artery: “FBJ”<br>Closure of subclavio-pulmonary connection: “FBK”<br>Connection to pulmonary artery from aorta: “FBL”<br>Closure of artificial aorto-pulmonary connection: “FBM”<br>Closure of fistula of pulmonary artery: “FBN”<br>Other operations on pulmonary artery and branches: “FBW”<br><br>Thoracic and thoracoabdominal aorta:<br>Repair of ascending aorta: “KFCA”<br>Repair of aortic arch: “KFCB”<br>Repair of descending aorta: “KFCC”<br>Repair of thoracoabdominal aorta: “KFCD”<br>Closure of acquired fistula from aorta: “KFCE”<br>Other operations on thoracic or thoracoabdominal aorta: “KFCW”<br><br>Operation of the heart and large intrathoracic vessels:<br>Repair of aortic valve stenosis: “KFMA”<br>Expansion of aortic ostium: “KFMB”<br>Repair of aortic valve for insufficiency: “KFMC”<br>Replacement of aortic valve: “KFMD”<br>Operation on people who have artificial heart valve: “KFMH”<br>Other operations on aortic valve: “KFMW”<br><br>Operation on arteries of aortic arch and branches:<br>Suture of arteries of aortic arch and branches: “KPAC”<br>Thrombectomy and embolectomy of arteries of aortic arch and branches: “KPAE”<br>Thrombendarterectomy of arteries of aortic arch and branches: “KPAF”<br>Operations for aneurysm of arteries of aortic arch and branches: “KPAG”		Thoracic and thoracoabdominal aorta:<br>”KFCA”, ”KFCB”, ”KFCC”, ”KFCD”, ”KFCE”, ”KFCW”<br><br>Operation of the heart and large intrathoracic vessels:<br>”FMA”, ”FMB”, ”FMC”, ”KFMD”, ”KFMH”, ”KFMW”<br><br>Operation on arteries of aortic arch and branches:<br>”KPAC”, ”KPAE”, ”KPAF”, ”KPAH”, ”KPAN”, ”KPAP”, ”KPAQ”, ”KPAW99”, ”KPAU74”

© 2020 Munk NE et al. *JAMA Network Open.*

eTable 1. Definition of Eligibility Criteria for Empagliflozin RCTs

RCTs ineligibility common ground	Definition in Danish medical databases	Databases	Codes
	Bypass from arteries of aortic arch and branches: “KPAH”<br>Plastic repair of arteries of aortic arch and branches: “KPAN”<br>Percutaneous plastic repair arteries of aortic arch and branches: “KPAP”<br>Insertion of stent into arteries of aortic arch and branches: “KPAQ”<br>Thrombectomy or embolectomy in bypass from carotid, subclavian or axillary artery: “KPAU74”<br>Other operations on arteries of aortic arch and branches: “KPAW99”<br><br>Operation on arteries of upper extremity:<br>Thrombectomy or embolectomy of arteries of upper extremity: “KPBE”<br>Thrombendarterectomy of arteries of upper extremity: “KPBF”<br>Bypass from arteries of upper extremity: “KPBH”<br>Plastic repair of arteries of upper extremity: “KPBN”<br>Percutaneous plastic repair of arteries of upper extremity: “KPBP”<br>Insertion of stent into arteries of upper extremity: “KPBQ”<br>Other operations on arteries of upper extremity: “KPBW”<br><br>Operation on suprarenal abdominal aorta and visceral arteries incl. kidneys:<br>“KPCE+F+H+N+P+Q”, “KPCW99”<br>Thrombectomy or embolectomy in bypass from suprarenal abdominal aorta and visceral arteries: “KPCU74”<br>Plastic repair in bypass from suprarenal abdominal aorta and visceral arteries: “KPCU82”<br>Percutaneous plastic repair in bypass from suprarenal abdominal aorta and visceral arteries: “KPCU83”<br>Insertion of stent into bypass from suprarenal abdominal aorta and visceral arteries: “KPCU84”<br><br>Operations on infrarenal abdominal aorta and iliac arteries and distal connections:		Operation on arteries of upper extremity: ”KPBE”, ”KPBF”, ”KPBH”, ”KPBN”, ”KPBP”, ”KPBQ”, ”KPBW”<br><br>Operation on suprarenal abdominal aorta and visceral arteries incl. kidneys: ”KPCE”, ”KPCF”, ”KPCH”, ”KPCN”, ”KPCP”, ”KPCQ”, ”KPCW99”, ”KPCW20”, ”KPCU74”, ”KPCU82”, ”KPCU83”, ”KPCU84”<br><br>Operations on infrarenal abdominal aorta and iliac arteries and distal connections: ”KPGF”, ”KPGH”, ”KPGN”, ”KPGP”, ”KPGQ”, ”KPGW99”,

© 2020 Munk NE et al. *JAMA Network Open.*

eTable 1. Definition of Eligibility Criteria for Empagliflozin RCTs

RCTs ineligibility common ground	Definition in Danish medical databases	Databases	Codes
	Infra renal abdominal aorta and iliac arteries: “KPDC+E+F+H+N+P+Q”, “KPGW99”<br>Operations on femoral artery with branches and connection to popliteal artery: ”KPEE+F+H+N+P+Q+W”<br>Popliteal artery:<br>KPFE+H+N+P+Q+W<br>Extra-anatomic bypass:<br>“KPGH20+21+22+23+30+31+40+99”<br>Repair after previous reconstruction of infrarenal abdominal aorta and iliac arteries and distal connections:<br>“KPDU74+82+83+84”<br>Repair after previous reconstruction of femoral artery with branches and connection to popliteal artery:<br>“KPEU74+82+83+84”<br>Repair after previous bypass from femoral or popliteal artery to infrapopliteal arteries and reconstruction of popliteal artery and arteries of lower leg and foot:<br>“KPFU74+82+83+84”<br><br>Extra-anatomic bypass: ”KPGH”<br>Thrombectomy or embolectomy of extra-anatomic bypass:<br>”KPGU74”<br>Percutaneous plastic repair of extra-anatomic bypass: ”KPGU83”<br>Insertion of stent into extra-anatomic bypass: ”KPGU84”<br>Other repair of extra-anatomic bypass: ”KPGU99”<br>Other extra-anatomic bypass operation: ”KPGW99”<br>Reoperation for thrombosis or embolism in surgery of peripheral vessels and lymphatic system:<br>”KPWG”<br><br>Coronary arteries incl. CABG and PTCA:<br>“KFNA, KFNB, KFNC, KFND, KFNE, KFNF, KFNG, KFNH, KFNW, KFLF”<br><br>Abdominal surgery:<br>Resection of eosophagus: ”KJCC”<br>Billroth I: ”KJDC00”<br>Billroth II: ”KJDC10”<br>Roux-en-Y reconstruction: ”KJDC20”		”KPGW20”, ”KPEE”, ”KPEF”, ”KPEH”, ”KPEN”, ”KPEP”, ”KPEQ”, ”KPEW”, ”KPFE”, ”KPFH”, ”KPFN”, ”KPFP”, ”KPFQ”, ”KPFW”, ”KPGH20”, ”KPGH21”, ”KPGH22”, ”KPGH23”, ”KPGH30”, ”KPGH31”, ”KPGH40”, ”KPGH99”, ”KPDU74”, ”KPDU82”, ”KPDU83”, ”KPDU84”, ”KPEU74”, ”KPEU82”, ”KPEU83”, ”KPEU84”, ”KPFU74”, ”KPFU82”, ”KPFU83”, ”KPFU84”,<br><br>Extra-anatomic bypass:<br>”KPGH” ”KPGU74”<br>”KPGU83” ”KPGU84”<br>”KPGU99” ”KPGW99”<br>”KPWG”<br><br>Coronary arteries incl. CABG and PTCA:<br>“KFNA”, ”KFNB”<br>“KFNC”, ”KFND”<br>”KFNE”, ”KFNF”<br>”KFNG”, ”KFNH”<br>”KFNW”, ”KFLF”<br><br>Abdominal surgery:<br>”KJCC”, ”KJDC00”, ”KJDC10”, ”KJDC20”, ”KJFB00”, ”KJFB40”, ”KJFB43”, ”KJFB46”, “KJLC”

eTable 1. Definition of Eligibility Criteria for Empagliflozin RCTs

RCTs ineligibility common ground	Definition in Danish medical databases	Databases	Codes
	Partial resection of small intestine: ”KJFB00”<br>Resection of transverse colon: “KJFB40”<br>Left hemicolectomy: “KJFB43”<br>Resection of sigmoid colon : “KJFB46”<br>Excision of pancreas: “KJLC”<br><br>Urinary system, male genital organs:<br>Total excision of kidney: ”KKAC”<br>Cystectomy: ”KKCC”<br>Total excision of prostate and seminal vesicles: ”KKEC”<br><br>Musculoskeletal surgery:<br>Exploratory procedures on hip and thigh: ”KNFA”<br><br>Medical conditions requiring removal to hospital/hospitalization for treatment (No outpatient setting). Within 4 weeks prior to first empagliflozin prescription<br><br>Certain infectious and parasitic diseases:<br>Tuberculosis: ”A15-A19”<br>Bubonic plague: ”A20”<br>Cutaneous anthrax: “A22”<br>Meningococcal infection: “A39”<br>Sepsis due to streptococcus: ”A40”<br>Other sepsis: ”A41”<br>Mosquito-borne viral encephalitis: ”A83”<br>Tick-borne viral encephalitis: ”A84”<br>Other viral encephalitis, not elsewhere classified: ”A85”<br>Viral meningitis: ”A87”<br>Yellow Fever: ”A95”<br>Dengue: ”A97”<br>Other viral hemorrhagic fevers, not elsewhere classified: ”A98”<br><br>Diseases of the nervous system:<br>Inflammatory diseases of the central nervous system: ”G00-G08”<br>Amyotrophic lateral sclerosis: ”G122G”<br>Cauda equina syndrome: ”G834”<br>Compression of brainstem: ”G935A”<br>Herniation of brainstem: ”G935C”<br>Cerebral oedema: ”G936”<br>Vascular myelopathies: ”G951A-G”		Urinary system, male genital organs: “KKAC”, ”KKCC”, ”KKEC”<br><br>Musculoskeletal surgery: “KNFA”<br><br>Medical conditions<br><br>Certain infectious and parasitic diseases: ”DA15-19”, ”DA20”,”DA22”, “DA39”, ”DA40”, ”DA41”, ”DA83”, ”DA84”,”DA85”, ”DA87”, ”DA95”, ”DA97”, ”DA98”<br><br>Diseases of the nervous system: ”DG00”, ”DG01”, ”DG02”, ”DG03”, ”DG04”, DG05”, ”DG06”, ”DG07”, ”DG08”, ”DG122G”, ”DG834”, ”DG935A”, ”DGG935C”, ”DG936”, ”DG951A”, DG951B”, ”DG951C”, ”DG951D”, DG951E”, DG951F”, DG951G”, ”DG952”<br><br>Diseases of the eye

© 2020 Munk NE et al. *JAMA Network Open.*

eTable 1. Definition of Eligibility Criteria for Empagliflozin RCTs

RCTs ineligibility common ground	Definition in Danish medical databases	Databases	Codes
	Cord compression: ”G952”<br><br>Diseases of the eye and adnexa:<br>Abscess of orbit: ”H05A”<br>Retinal detachment with retinal break: ”H33”<br>Central retinal artery occlusion: ”H341”<br>Within 2 weeks: (before first empagliflozin prescription): narrow-angle glaucoma: ”H402”<br><br>Diseases of the circulatory system:<br>Aortic aneurysm and dissection: ”DI71”<br>Hypertensive heart and renal disease: ”I13”<br>Pulmonary embolism with mention of acute cor pulmonale: ”I26”<br>Disease of pericardium, unspecified: ”I319A”<br>Acute and subacute infective endocarditis: I33+38+39<br>Atherosclerosis of arteries of extremities: ”I702A”<br>Arterial embolism and thrombosis: ”I742-I745”<br><br>Diseases of the respiratory system:<br>Acute respiratory distress syndrome: “J809B+C”<br>Gangrene and necrosis of lung: ”J850”<br>Pneumothorax: ”J930”<br><br>Diseases of the digestive system:<br>Idiopathic acute pancreatitis: “K85”<br>Acute peritonitis: “K650”<br>Esophageal varices: “I85”<br>Acute vascular disorders of intestine: “K550”<br>Alcoholic liver disease: ”K70”<br>Endocrine and metabolic diseases:<br>Thyroid crisis or storm: ”E055”<br>Nondiabetic hypoglycaemic coma: ”E15”<br>Encephalopathy after hypoglycemic coma: ”E161B”<br>Addisonian crisis: “E27.2”<br>Type 2 diabetes gangrene: ”E11.5C”<br>Wernicke encephalopathy: ”E51.2”<br>Cystic fibrosis: ”E84”<br>Acidosis: ”E872”		and adnexa: ”DH05A”, ”DH33”, ”DH341”, ”DH402”<br><br>Diseases of the circulatory system: ”DI71”, ”DI132”, ”DI26”, ”DI319A”, ”DI33”, ”DI38”, ”DI39”, “DI702A”, “DI742”, ”DI743”, ”DI744”, ”DI745”<br><br>Diseases of the respiratory system: ”DJ809B”, ”DJ809C”, ”DJ850”, ”DJ930”<br><br>Diseases of the digestive system: ”DK85”, ”DK650”, ”DI85”, “DK550”, ”DK701”, ”DK702”, ”DK703”, ”DK704”, “DE055”, “DE15”, “DE161B”, “DE272”, “DE115C”, ”DE512”, ”DE84”, ”DE872”, ”DM860”,”DM87”

© 2020 Munk NE et al. *JAMA Network Open.*

eTable 1. Definition of Eligibility Criteria for Empagliflozin RCTs

RCTs ineligibility common ground	Definition in Danish medical databases	Databases	Codes
	Acute hematogenous osteomyelitis: ”M860”<br>Idiopathic aseptic necrosis of bone: ”M87”		

© 2020 Munk NE et al. *JAMA Network Open.*

**eTable 2. Real-World Empagliflozin Initiators Who Would Have Been Excluded From RCTs and Changes in Their Glycated Hemoglobin Levels**					
**Eligibility criteria for RCT participation**	**Real-world initiators that would have been excluded from RCTs**		**Mean HbA_{1c} reduction**	**HbA_{1c} before empagliflozin initiation**	**HbA_{1c} months after empagliflozin initiation**
	n	%	%	%	%
HbA_{1c} level <7%	715	10.2	0.10 (0.04, 0.17)	6.62 (6.60, 6.65)	6.74 (6.68, 6.63)
HbA_{1c} level >10%	1,057	15.0	-2.45 (-2.58, -2.32)	11.09 (11.03, 11.15)	8.52 (8.41, 8.63)
Kidney disease	92	1.3	-0.55 (-0.86, -0.23)	8.40 (8.16, 8.64)	7.87 (7.61, 8.13)
Cancer	308	4.4	-0.78 (-0.96, -0.60)	8.38 (8.24, 8.53)	7.65 (7.50, 7.80)
Blood dyscrasias	455	6.5	-0.75 (-0.90, -0.60)	8.32 (8.20, 8.45)	7.64 (7.52, 7.76)
Anti-obesity drugs	17	0.2	-0.81 (-1.65, 0.03)	8.63 (7.88, 9.45)	7.76 (7.10, 8.48)
Systemic glucocorticoids	235	3.3	-1.05 (-1.26, -0.83)	8.40 (8.24, 8.57)	7.46 (7.32, 7.61)
Mental incapacity	129	1.8	-1.22 (-1.54, -0.90)	8.83 (8.54, 9.13)	7.69 (7.42, 7.97)
Metformin, sulphonyl-urea or pioglitazone	589	8.4	-0.74 (-0.88, -0.61)	8.53 (8.41, 8.65)	7.79 (7.67, 7.91)
GLP-1RA, DPP-4i, SGLT2i* or meglitinide	1366	19.4	-0.85 (-0.94, -0.77)	8.46 (8.39, 8.53)	7.65 (7.59, 7.72)
Clinical conditions jeopardizing initiators' safety during trial	141	2.1	-1.0 (-1.4, -0.6)	8.52 (8.26, 8.78)	7.58 (7.34, 7.83)
Liver disease	71	1.0	-1.16 (-1.69, -0.63)	8.59 (8.22, 8.98)	7.57 (7.21, 7.95)
Alcohol or drug abuse	8	0.1	-0.87 (-2,21, 0.87)	8.82 (8.07, 9.64)	7.88 (6.51, 9.55)
Trial ineligible	3,878	55.1	-1.01 (-1.07, -0.95)	8.60 (8.55, 8.66)	7.67 (7.63, 7.72)
Trial eligible	3,156	44.9	-0.78 (-0.82, -0.74)	8.26 (8.24, 8.29)	7.48 (7.44, 7.51)
Total initiators	7,034	100	-0.91 (-0.94, -0.87)	8.45 (8.42, 8.48)	7.58 (7.55, 7.61)

Caption: *Other than empagliflozin. Data are presented as mean (95% CI) HbA_{1c} (%). Abbreviations: HbA_{1c}, glycated haemoglobin; DPP4i, dipeptidyl peptidase-4 inhibitors; GLP1-RA, glucagon-like peptide-1 receptor agonists; SGLT2, sodium-glucose cotransporter 2 inhibitor; SU, sulphonylureas.

© 2020 Munk NE et al. *JAMA Network Open.*
